# Predictors of residual disease after debulking surgery in advanced stage ovarian cancer

**DOI:** 10.3389/fonc.2023.1090092

**Published:** 2023-01-24

**Authors:** Farnoosh Abbas-Aghababazadeh, Naoko Sasamoto, Mary K. Townsend, Tianyi Huang, Kathryn L. Terry, Allison F. Vitonis, Kevin M. Elias, Elizabeth M. Poole, Jonathan L. Hecht, Shelley S. Tworoger, Brooke L. Fridley

**Affiliations:** ^1^ Department of Biostatistics & Bioinformatics, H. Lee Moffitt Cancer Center and Research Institute, Tampa, FL, United States; ^2^ University Health Network, Princess Margaret Cancer Center, Toronto, ON, Canada; ^3^ Department of Obstetrics and Gynecology, Brigham and Women’s Hospital and Harvard Medical School, Boston, MA, United States; ^4^ Department of Cancer Epidemiology, H. Lee Moffitt Cancer Center and Research Institute, Tampa, FL, United States; ^5^ Department of Medicine, Channing Division of Network Medicine, Brigham and Women’s Hospital, Boston, MA, United States; ^6^ Sanofi Genzyme, Global Medical Affairs, Cambridge, MA, United States; ^7^ Department of Pathology, Beth Israel Deaconess Medical Center, Boston, MA, United States

**Keywords:** ovarian cancer, debulking, residual disease, prediction model, immunohistochemistry, tissue microarray

## Abstract

**Objective:**

Optimal debulking with no macroscopic residual disease strongly predicts ovarian cancer survival. The ability to predict likelihood of optimal debulking, which may be partially dependent on tumor biology, could inform clinical decision-making regarding use of neoadjuvant chemotherapy. Thus, we developed a prediction model including epidemiological factors and tumor markers of residual disease after primary debulking surgery.

**Methods:**

Univariate analyses examined associations of 11 pre-diagnosis epidemiologic factors (n=593) and 24 tumor markers (n=204) with debulking status among incident, high-stage, epithelial ovarian cancer cases from the Nurses’ Health Studies and New England Case Control study. We used Bayesian model averaging (BMA) to develop prediction models of optimal debulking with 5x5-fold cross-validation and calculated the area under the curve (AUC).

**Results:**

Current aspirin use was associated with lower odds of optimal debulking compared to never use (OR=0.52, 95%CI=0.31-0.86) and two tissue markers, ADRB2 (OR=2.21, 95%CI=1.23-4.41) and FAP (OR=1.91, 95%CI=1.24-3.05) were associated with increased odds of optimal debulking. The BMA selected aspirin, parity, and menopausal status as the epidemiologic/clinical predictors with the posterior effect probability ≥20%. While the prediction model with epidemiologic/clinical predictors had low performance (average AUC=0.49), the model adding tissue biomarkers showed improved, but weak, performance (average AUC=0.62).

**Conclusions:**

Addition of ovarian tumor tissue markers to our multivariable prediction models based on epidemiologic/clinical data slightly improved the model performance, suggesting debulking status may be in part driven by tumor characteristics. Larger studies are warranted to identify those at high risk of poor surgical outcomes informing personalized treatment.

## Introduction

Ovarian cancer has a 5-year survival <50%, as most cases are diagnosed at late stages (1). Optimal debulking with no or minimal residual disease during cytoreductive surgery is predictive of survival (2). Randomized trials showed 20% higher risk of death and 25% higher risk of progression among patients with residual tumor >10mm versus 1-10mm (3). Identifying women who may have poor surgical outcomes is critical to defining appropriate treatment, including use of neo-adjuvant chemotherapy to reduce tumor burden (4).

Studies of preoperative predictors of suboptimal debulking had not led to a reproducible model. For example, findings are mixed regarding the predictive value of preoperative blood CA125 (5) and radiologic features from computed tomography scans (6). Data is limited regarding epidemiologic factors related to debulking, with mixed results for obesity and hormone therapy (7, 8). Furthermore, prior studies observed that tumor molecular characteristics can predict residual disease, identifying gene expression signatures in migration, invasion, and stromal activation pathways (9–13). However, no studies to date have assessed a comprehensive prediction model for debulking outcomes. Thus, our objectives were to identify epidemiological characteristics and tumor markers associated with residual disease and build a prediction model of optimal debulking status after primary debulking surgery in treatment naïve, advanced stage, invasive epithelial ovarian cancer patients.

## Materials and methods

The Nurses’ Health Study (NHS) is a prospective cohort study established in 1976 enrolling 121,000 female nurses ages 30-55 years from 11 US states (14). NHSII was established in 1989 enrolling 116,429 female nurses ages 25-42 years from 14 US states (15). Women provided demographic, lifestyle, reproductive, and medical information biennially. Self-reported ovarian cancer diagnosis were confirmed by pathology report review (94%) or linkage to tumor registries. A gynecologic pathologist abstracted information from pathology and surgical reports on stage, histology, grade, and residual tumor (optimal debulking: residual tumor <2cm; suboptimal debulking: residual tumor ≥2cm; unknown). This definition was used because most cases were diagnosed when this threshold was used to define optimal debulking. The study protocol was approved by the institutional review boards of the Brigham and Women’s Hospital and Harvard T.H. Chan School of Public Health, and those of participating registries as required.

The New England Case-Control Study (NECC) is a population-based case control study of ovarian cancer enrolling over three phases (1992-1997, 1998-2002, 2003-2008) from New Hampshire and Eastern Massachusetts (16); 2,203 (71%) of eligible cases identified using area hospital registries agreed to participate. Participants completed in-person interviews on demographics, lifestyle, reproductive factors, and medical history one year prior to ovarian cancer diagnosis. Surgical and pathological reports were reviewed to confirm diagnosis and abstract stage, histology, and grade. Optimal vs. suboptimal debulking was defined as residual disease <1cm vs. ≥1cm or unknown. The study was approved by the Institutional Review Board of Brigham and Women’s Hospital, Boston, Massachusetts and Dartmouth College, Hanover, New Hampshire.

Epidemiologic factors included age (years, continuous), body mass index (BMI; kg/m^2^, continuous), smoking (never, current, former), duration of smoking (pack-years, continuous), oral contraceptive use (OC; never, ever), parity (0, 1, 2+), menopausal status (premenopausal, postmenopausal never used hormone therapy [HT], postmenopausal ever used HT), aspirin use (never, current, past), family history of breast or ovarian cancer (no, yes), and history of hysterectomy, tubal ligation or Cesarean section (ever, never) at least one year before diagnosis.

Details on ovarian tumor block collection has been described previously (17). We retrieved formalin fixed paraffin embedded (FFPE) blocks with primary ovarian tumor from 631 invasive cases (330 NHS, 86 NHSII, 215 NECC). Blocks were reviewed for histology, invasiveness, and grade by a gynecologic pathologist, using 2014 WHO diagnostic criteria, circling areas of tumor for tissue microarrays (TMA) with two 1.0mm or three 0.6mm cores per case (17). We used histology, invasiveness, and grade from the slide review and record abstraction otherwise.

Immunohistochemistry (IHC) staining was performed on TMA slides within two weeks of sectioning (**Supplementary Table S1**). IHC markers, except pSMAD2/3, were evaluated by a gynecologic pathologist based on the proportion of cells staining positive (0-3+) or mutant/abnormal or wild type (TP53). For pSMAD 2/3, we assessed H-score (18) based on intensity and percent staining area, calculated *via* Definiens Tissue Studio v4.2 suite (Definiens Inc, Germany) with scans from Aperio™ ScanScope AT2 or AT Turbo (Leica Biosystems, Vista, CA) at Moffitt Cancer Center. All IHC markers were evaluated in the tumor epithelial component. For this study, we included IHC markers that have previously been reported to be associated with debulking status (i.e. ADH1B, COL11A1, CXCL14, FABP4, FAP, POSTN, pSmad2/3) and other IHC markers in which data were generated as part of pprior studies (i.e. ADRB2 (19), CD163 (20), CD68 (20), PTGS1 (20), PTGS2 (20), ESR1 (17, 21), ESR2n (22), MAPK (23), MUC1, MUC16, TP53 (23), PGR (17, 21), STAT1, VDRc, VDRn).

### Statistical analysis

#### Imputation

Imputation of missing IHC scores was conducted using *k*-Nearest Neighbors (*k*NN; k=5) since some markers were missing in selected TMAs (**Supplementary Figure S1**). Imputed data had more symmetrical distribution and slightly higher median values versus observed data (**Supplementary Figure S2**).

#### Clinical and tissue biomarker predictors of debulking status

Mean and standard deviation (SD) for continuous variables and frequencies and percentage for categorical variables were used to summarize the predictors. Tissue markers were treated continuously. The primary outcome was coded as optimal versus suboptimal debulking. Analyses were conducted including all cases and restricted to type II ovarian tumors (high-grade serous, endometrioid, mixed or poorly differentiated, Transitional/Brenner, carcinosarcoma) (24). Logistic regression was used to examine the association of epidemiologic characteristics and normalized IHC scores with debulking status, adjusting for study site (NECC, NHS/NHSII). Cox proportional hazards regression was used to assess the relationship of debulking status with overall survival to ensure the validity of our debulking measure.

#### Prediction modeling

Bayesian model averaging (BMA) with logistic regression was used to develop the prediction model for debulking status with only additive effects (i.e., no interaction effects) using the BMA R package (25). BMA was fit using five 5-fold cross-validations (CV), resulting in different training and test sets for each run to improve estimated performance. For each predictor, we present the average posterior mean (APM) and average posterior SD (APSD) across the five 5-fold CV. The receiver operating characteristics curve was calculated from the posterior probabilities. For each fold, we evaluated the discriminatory accuracy using the area under curve (AUC) and calculated the mean for each CV, and the overall AUC by averaging the average AUCs of the 5 CVs and computing the associated standard deviation (SD). We conducted BMA for epidemiological/clinical variables with debulking status (n=593). Then, after creating a single predictive score from the epidemiologic variables, we conducted BMA adding the tumor marker data. Analyses were performed using R, version 4.0.2.

## Results

**Figure 1:**
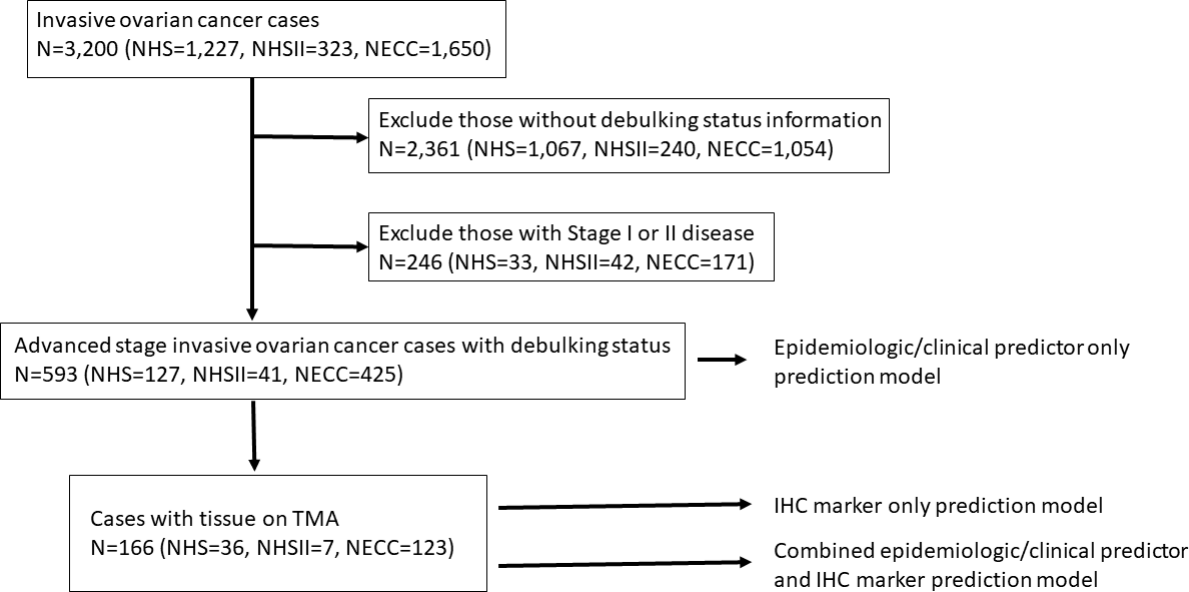
Flow chart of the exclusion criteria and study population included in the various prediction modeling.

In NHS/NHSII, of the 1,550 incident invasive ovarian cancer cases (1,227 in NHS and 323 in NHSII) diagnosed from 1976-2017, we excluded those with unknown debulking status (n=1,307; 1,067 in NHS and 240 in NHSII) and stage I or II disease (n=75, 33 in NHS and 42 in NHSII). In NECC, of the 1,650 invasive epithelial ovarian cancer cases, we excluded those who did not have information on debulking status (n=1,054) and stage I or II disease (n=171) (**Figure 1**). As a result, the epidemiologic model included 593 invasive epithelial ovarian cancer cases (NHS=127, NHSII=41, NECC=425), of which 464 (78%) were optimally debulked (type II n=537 and 419 optimally debulked; **Table 1**). Average age at diagnosis was 56.9 years (SD 12.6) with the majority (>90%) being type II tumors. Characteristics of cases by debulking status were similar (**Supplementary Table S2**). Cases with optimal versus suboptimal debulking had better overall survival (all: HR=0.60, 95%CI=0.48-0.75; type II: HR=0.63, 95%CI=0.50-0.80). Current vs. never aspirin use was significantly associated with lower odds of optimal debulking (OR=0.52, 95%CI=0.31-0.86), which remained significant for type II tumors (OR=0.47, 95%CI=0.28-0.81; **Supplementary Table S3**).

**Table 1:** Association between demographic/clinical characteristics and debulking status (1=optimally debulked, 0 = sub-optimally debulked) among advanced stage invasive epithelial ovarian cancer cases in NHS/NHSII/NECC.

	Total (n=593)		Optimally debulked (n=464)		Sub-optimally Debulked (n=129)	OR (95% CI) optimally vs. sub-optimally debulked
**Age at diagnosis (years),** Mean (SD)	56.9 (12.6)		56.9 (12.6)		56.7(12.8)	1.00 (0.99 to 1.02)
**BMI (kg/m^2^),** Mean (SD)	26.2 (5.7)		26.2 (5.7)		26.1 (5.6)	1.00 (0.97 to 1.04)
**Smoking status**, n (%)						
never	270 (45.5%)		213 (45.9%)		57 (44.2%)	1 (ref.)
current	87 (14.7%)		64 (13.8%)		23 (17.3%)	0.74 (0.43 to 1.32)
former	236 (39.8%)		187 (40.3%)		49 (38.0%)	1.02 (0.67 to 1.57)
**Smoking (pack-years),** Mean (SD)	11.7 (19.6)		11.2 (19.2)		13.5 (21.0)	1.00 (0.99 to 1.00)
**Aspirin,** n (%)						
never	391 (65.9%)		317 (68.3%)		74 (57.4%)	1 (ref.)
current	93 (15.7%)		64 (13.8%)		29 (22.5%)	0.52 (0.31 to 0.86)
past	109 (18.4%)		83 (17.9%)		26 (20.2%)	0.75 (0.45 to 1.25)
**Oral Contraceptive use**, n (%)						
never	294 (49.6%)		224 (48.3%)		70 (54.3%)	1 (ref.)
ever	299 (50.4%)		240 (51.7%)		59 (45.7%)	1.27 (0.86 to 1.88)
**Parity,** n (%)						
0	122 (20.6%)		97 (20.9%)		25 (19.4%)	1 (ref.)
1	60 (10.1%)		51 (11.0%)		9 (7.0%)	1.46 (0.65 to 3.52)
2 +	411 (69.3%)		316 (68.1%)		95 (73.6%)	0.86 (0.51 to 1.39)
**Menopausal status,** n (%)						
premenopausal	155 (26.1%)		120 (25.9%)		35 (27.1%)	1 (ref.)
postmenopausal never used PMH	242 (40.8%)		190 (40.9%)		52 (40.3%)	1.07 (0.65 to 1.73)
postmenopausal ever used PMH	196 (33.1%)		154 (33.2%)		42 (32.6%)	1.07 (0.64 to 1.78)
**Family history of breast or ovarian cancer**, n (%)						
No	512 (86.3%)		396 (85.3%)		116 (89.9%)	1 (ref.)
Yes	81 (13.7%)		68 (14.7%)		13 (10.1%)	1.53 (0.84 to 2.99)
**History of surgery ^a^**, n (%)						
Ever	206 (34.7%)		159 (34.3%)		47 (36.4%)	1 (ref.)
Never	387 (65.3%)		305 (65.7%)		82 (63.6%)	1.10 (0.73 to 1.65)
**Tumor Type ^b^**, n (%)						
type 1	56 (9.4%)		193 (27.7%)		11 (8.5%)	1 (ref.)
type 2	537 (90.6%)		504 (72.3%)		118 (91.5%)	0.87 (0.42 to 1.67)

BMI, Body mass index; CI, Confidence interval; NHS, Nurses’ Health Study; NHSII, Nurses’ Health Study II; NEC, New England Case-Control Study; OR, Odds ratio; PMH, postmenopausal hormone use.

All models were adjusted for study sites (NHS/NHSII and NEC). Odds ratios represent the odds of optimally debulked surgery.

(a) Cases with history of hysterectomy or tubal ligation or Cesarean section.

(b) Type 1 tumors: low-grade serous, mucinous, endometrioid, clear cell, low grade mixed; type 2 tumors: high-grade serous or poorly differentiated, Transitional/Brenner, Carcinosarcoma, high grade mixed.

**Figure 2:**
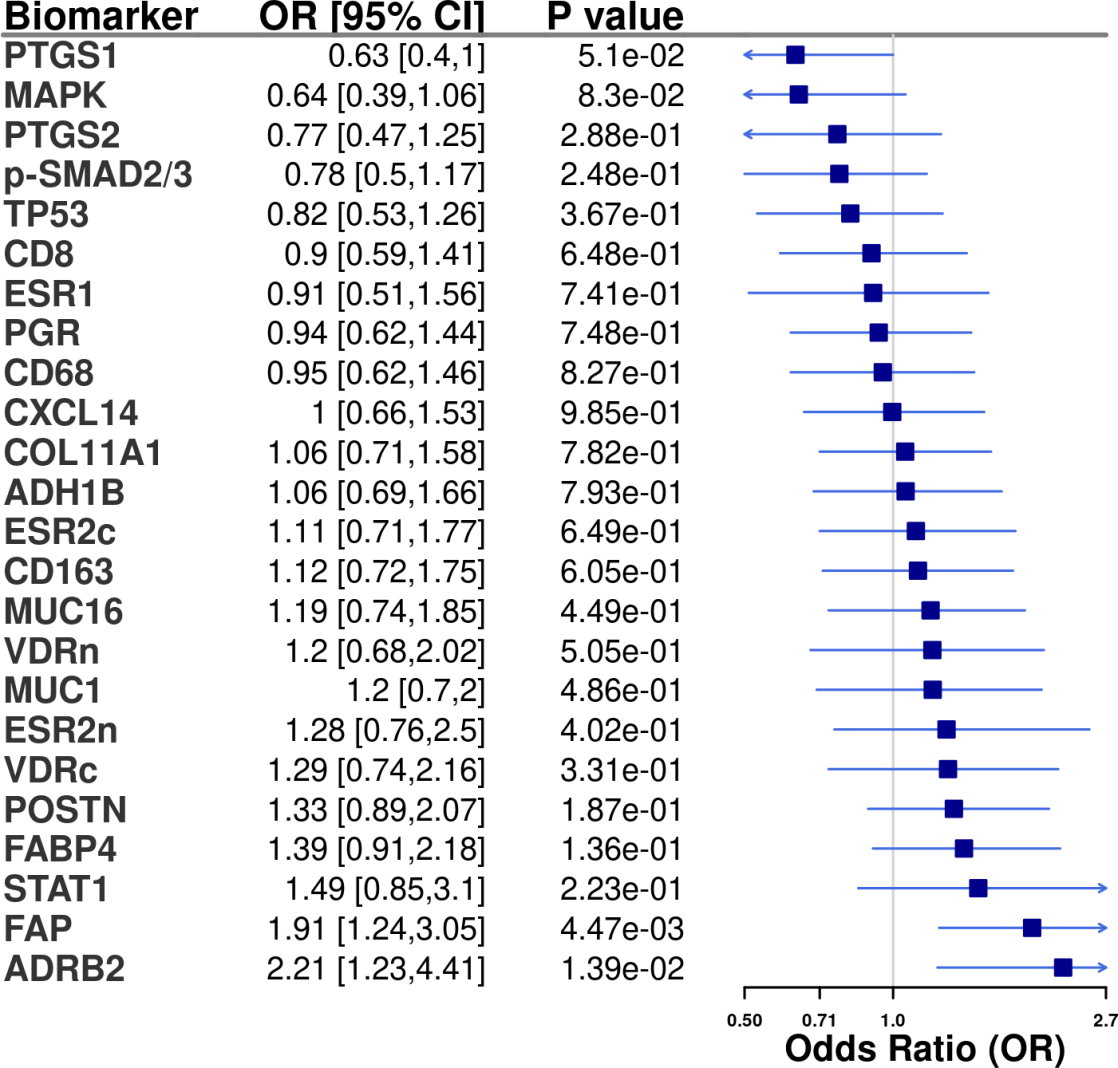
Tissue marker associations (odds ratio [OR] and 95% confidence intervals [CI]) with optimal debulking status among advanced stage invasive epithelial ovarian cancer cases in NHS/NHSII/NECC (n=166). All models were adjusted for study (NHS/NHSII and NEC). In the logistic regression models 1 = optimally debulked and 0 = sub-optimally debulked.

Tissue IHC markers were available in 166 cases with data on debulking status, of which 135 (81%) were optimally debulked. Distribution of clinical and epidemiologic characteristics were similar to all cases (**Supplementary Table S4**; **Supplementary Table S5**). ADRB2 (OR=2.21, 95%CI=1.23-4.41) and FAP (OR=1.91, 95%CI=1.24-3.05) were associated with optimal debulking (**Figure 2**). Results were similar for type II tumors (**Supplementary Figure S3**).

### Prediction modeling of optimal debulking status using epidemiologic and clinical predictors

**Figure 3:**
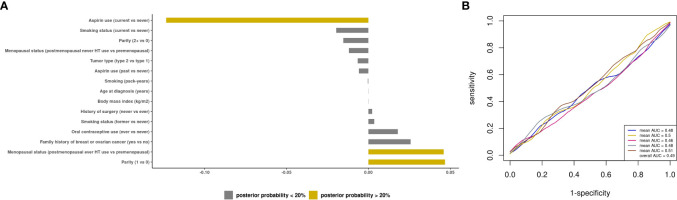
Prediction modeling of optimal debulking status using epidemiologic and clinical predictors. Average posterior means and associated average posterior probabilities of epidemiologic and clinical predictors being selected in the final prediction model of optimal debulking status and area under the curve (AUC) of the prediction models using Bayesian model averaging among invasive epithelial ovarian cancer cases (n=593) in NHS/NHSII/NEC. We assessed the posterior probability of 11 predictors for possible selection in the final model and conducted five 5-fold cross-validations. The bar chart **(A)** presents the average posterior mean across the 25 models that were run in total; grey bars denote predictors with average posterior probabilities <20% and yellow bars denote predictors with average posterior probability ≥ 20%. **(B)** presents the five average AUCs from the five 5-fold cross-validations and in the legend overall AUC, which is the average of the average AUCs from the 5-fold CV, is presented. Type 2 tumors include high-grade serous or poorly differentiated, Transitional/Brenner, Carcinosarcoma, high grade mixed histology.

We first sought to develop a prediction model using epidemiologic and clinical predictors only. The BMA results for all models are presented in **Supplemental File 1**. Three predictors had a posterior effect probability ≥20% for selection into the prediction model: current vs. never aspirin use (APM=-0.12, APSD=0.21), parity=1 vs. nulliparous (APM=0.05, APSD=0.16), and postmenopausal ever HT vs. premenopausal (APM=0.05, APSD=0.12 (**Figure 3A**). For type II tumors, 3 predictors were identified (current aspirin use, APM=-0.18, APSD=0.28; smoking pack-years, APM=-0.001, APSD=0.002, and menopausal status/ever HT use, APM=0.04, APSD=0.15; **Supplementary Figure S4**). The mean AUC of 0.49 (SD 0.02) for all invasive ovarian cancer (**Figure 3B**) and 0.53 (SD 0.03) for type II tumors (**Supplementary Figure S4**).

### Prediction modeling of optimal debulking status using tissue markers

**Figure 4:**
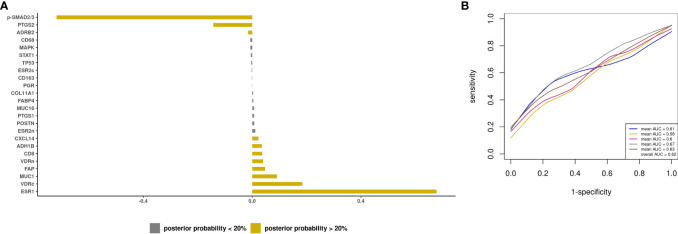
Prediction modeling of optimal debulking status using tissue markers. Average posterior means and associated average posterior probabilities of tissue markers being selected in the final prediction model of optimal debulking status and area under the curve (AUC) of the prediction models using Bayesian model averaging among invasive epithelial ovarian cancer cases (n=166) in NHS/NHSII/NEC. We assessed the posterior probability of 24 tissue marker predictors for possible selection in the final model and conducted five 5-fold cross-validations. The bar chart **(A)** presents the average posterior mean across the 25 models that were run in total; grey bars denote predictors with average posterior probabilities <20% and yellow bars denote predictors with average posterior probability ≥ 20%. **(B)** presents the five average AUCs from the five 5-fold cross-validations and in the legend overall AUC, which is the average of the average AUCs from the 5-fold CV, is presented.

Next, we developed a prediction model using tissue IHC markers only. There were 8 biomarkers with posterior effect probability of ≥20% including ESR1 and CD8+ T cells, which were associated with higher odds of optimal debulking (APM from -0.72 to 0.04), while p-SMAD2/3, PTGS2, and ADRB2 had lower odds of optimal debulking (APM of -0.72 to -0.02) (**Figure 4A**). For type II tumors, there were 7 biomarkers with positive posterior means (APM of 0.02 to 0.2) and 6 biomarkers with negative posterior means (APM of -0.14 to -0.02) (**Supplementary Figure S5**). These IHC markers resulted in a mean AUC of 0.62 (SD 0.03) for advanced stage invasive cases, and 0.47 (SD 0.1) for type II tumors (**Figures 4B**; **Supplementary Figure S5**).

### Combined prediction model with epidemiologic variables and tissue biomarkers

**Figure 5:**
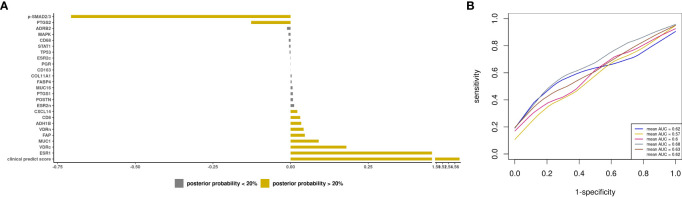
Prediction modeling of optimal debulking status using epidemiologic/clinical predictors and tissue markers. Average posterior means and associated average posterior probabilities of epidemiologic/clinical predictors and tissue markers being selected in the final prediction model of optimal debulking status and area under the curve (AUC) of the prediction models using Bayesian model averaging among invasive epithelial ovarian cancer cases (n=166) in NHS/NHSII/NEC. We assessed the posterior probability of 24 tissue markers in addition to our base model of clinical predict score, which included epidemiologic/clinical variables that had ≥ 20% posterior probability of being selected in the final prediction model of optimal debulking status (i.e. aspirin, parity, and menopausal status), for possible selection in the final model and conducted five 5-fold cross-validations. The bar chart **(A)** presents the average posterior mean across the 25 models that were run in total; grey bars denote predictors with average posterior probabilities <20% and yellow bars denote predictors with average posterior probability ≥ 20%. **(B)** presents the five average AUCs from the five 5-fold cross-validations and in the legend overall AUC, which is the average of the average AUCs from the 5-fold CV, is presented.

Lastly, we developed a prediction model including both epidemiologic/clinical predictors and tissue biomarkers. Among the subset of cases with biomarker data, the model with only the clinical prediction score had a mean AUC of 0.58 (SD 0.07) for all cases and 0.62 (SD 0.01) for type II tumors. When tissue biomarkers were added, positive posterior mean was observed for 8 markers including ESR1 (APM=0.67, APSD=0.38) and CD8+ T cells (APM=0.03, APSD=0.12) and a negative posterior mean for p-SMAD2/3 (APM=-0.71, APSD=0.35) and PTGS2 (APM=-0.13, APSD=0.23) (**Figure 5A**). The clinical prediction score had an average posterior mean of 1.58 (APSD=7.94). The resultant mean AUC was 0.62 (SD 0.04) (**Figure 5B**). We observed similar results for type II tumors, although the model resulted in an AUC=0.47 (SD 0.1; **Supplementary Figure S6**).

## Discussion

We simultaneously examined a wide range of potential epidemiologic and molecular predictors of optimal debulking in advanced stage invasive ovarian cancer patients undergoing primary debulking surgery in a population-based study. Relatively few epidemiologic predictors were identified, and they did not have predictive capacity. However, four tumor markers (POSTN, pSMAD2/3, CXCL14, ADH1B, FAP) that have previously been reported to predict suboptimal debulking were selected in our model, although only p-SMAD2/3 was in the same direction but with lower discriminatory performance compared to prior reports. Seven other tumor markers predicted optimal debulking. However, the multivariable prediction model showed discriminatory performance that is not clinically actionable.

Several studies have previously identified ovarian tumor tissue markers that predict debulking status (9–13), with some studies reporting high discriminatory performance (11–13). One recent study sought to validate 20 previously reported molecular signatures using gene expression data and all combinations resulted in poor performance with AUC < 0.65 (10), which is consistent with our observation of poor performance for a tissue marker only prediction model. Notably, the direction of association reported in prior gene expression studies often showed opposite associations using IHC markers as in our study (11, 12). This may be due to the use of protein markers, which do not always correlate with gene expression, and the use of a population-based sample in our study. It is unclear if the protein markers do not provide the same predictive capacity as gene expression or if the high dimensionality of gene expression data is led to overfitting of results. Overall, our work and others support that biologic features of the tumor may differ between optimally and sub-optimally debulked tumors.

Even though aspirin use has been associated with a lower ovarian cancer risk (26), pre-diagnostic current aspirin use was associated with decreased odds of optimal debulking. This is inconsistent with reported null associations between pre-diagnostic aspirin use and ovarian cancer survival (27). Complications during surgery may be one reason for this observation (10) as aspirin users may be more likely to develop hemorrhage-related surgical complications. Parous women had increased odds of optimal debulking, in line with studies reporting modest inverse associations between parity and ovarian cancer survival or risk of aggressive disease (28, 29). HT use also increased odds of optimal debulking, consistent with an international case-control consortium reporting that pre-diagnosis HT was associated with lower risk of having macroscopic residual disease and improved survival among postmenopausal patients (8). Interestingly, pre-diagnostic smoking was associated with decreased odds of optimal debulking among type II tumors, which is consistent its association with worse survival (30) and increased risk of aggressive rapidly fatal disease in high-grade serous tumors (29). Smoking increases systemic inflammation (31) and also has been reported to accelerate migration and invasion of ovarian cancer cells, promoting progression and metastasis (32), which may result in tumors that are more likely to be sub-optimally debulked. Future work, with larger sample sizes, should further explore these relationships to validate our observations.

With respect to the biomarkers, we identified new potential predictors of debulking, including CD8+ T cells and ESR1, which increased odds of optimal debulking. CD8+ T cell infiltration has been associated with improved ovarian cancer survival (33). It is possible that an immune-activated tumor microenvironment results in tumors that are easier to surgically resect, possibly by reducing metastatic spread (34). ESR1 expression has been associated with improved ovarian cancer survival and lower risk of macroscopic residual disease in endometrioid tumors (35). Conversely, PTGS2 and ADRB2 were associated with lower odds of optimal debulking. Both PTGS2, which drives prostaglandin synthesis in the tumor microenvironment, and ADRB2 activation can increase cell migration, enhance cell survival (36), and promote cancer growth and metastasis (37). One study reported that concurrent increased expression of ADRB2 and PTGS2 in ovarian cancer was associated with poor survival (37), suggesting activation of this axis should be explored as a biological pathways driving disease spread, leading to residual disease. Additional large-scale, population-based studies are needed to evaluate the biologic differences between tumors that were optimally versus sub-optimally debulked and evaluate and validate the predictive capacity of these biomarkers above that of clinical and epidemiologic measures.

The strength of our study is that we had detailed epidemiologic/clinical and tumor tissue marker data and applied BMA to develop a multivariable prediction model. Limitations include the number of sub-optimally debulked patients and different definitions of optimal debulking across studies due to change in the criteria over time. We also were unable to evaluate complete cytoreductive surgery, although many prior studies used 1cm of residual disease as the cutoff for defining optimal debulking status. In NHS/NHSII and NECC, many ovarian cancer cases were missing data on debulking status (84% in NHS/NHSII; 64% in NECC), which may not be missing at random and possibly biased the observed results. However, the distribution of epidemiologic factors in our analytic sample were similar between those with and without data on debulking status, suggesting a representative sample. Furthermore, there could be reporting bias of debulking status particularly as many women in NHS/NHSII were treated in community settings, which may explain the high percentage of optimally debulked cases (83%) in our study compared to prior studies (range~40%-90%) (38), although debulking status was strongly associated with survival in our population. In NECC, nearly all cases received surgical care by subspecialist gynecologic oncologists at tertiary academic hospitals, likely leading to improved surgical outcomes (39). Our study was limited by not have an independent validation cohort to validate our prediction models, so conducted internal validation using 5x5-fold cross-validation. We did not have a measure of surgical skills by individual surgeons, which may vary widely due to the population-based nature of our study, or detailed laparoscopic data on tumor spread, both of which have been shown to be related to debulking status. While there were some IHC markers that are known to be more present in the stromal component (e.g. POSTN, COL11A1), our ovarian TMA was created to maximize the tumor epithelial tissue and the IHC scoring was based on the expression in the tumor epithelial compartment. Further studies are necessary to evaluate protein expression in the stromal compartments. Finally, we could not study laparoscopy-based scores, which have reported discriminatory performance ranging widely (AUCs~0.69-0.98), depending on outcome definition (complete and/or optimal cytoreduction) and the proportion of cases undergoing neo-adjuvant chemotherapy (40). Adding molecular factors to existing laparoscopy-based scores could enhance discriminatory ability in the primary debulking setting, which is most critical time to determine the need for neoadjuvant chemotherapy.

Overall, combining information on ovarian tumor tissue markers and epidemiologic/clinical data led to the best model performance, although it is not yet clinically actionable. Our results further support that debulking status may be in part driven by tumor characteristics. Future studies are warranted to validate our findings and integrate these variables into currently used clinical models based on disease spread to identify those at high risk of poor surgical outcomes, which will inform personalized treatment for ovarian cancer.

## Data availability statement

The datasets presented in this article are not publicly available because of participant confidentiality and privacy concerns. Further information including the procedures to obtain and access data from the Nurses’ Health Studies is described at https://www.nurseshealthstudy.org/researchers (contact email: nhsaccess@channing.harvard.edu). The NEC data that support the findings of this study are available upon request and review by study leadership.

## Ethics statement

The study protocol was approved by the institutional review boards of the Brigham and Women’s Hospital and Harvard T.H. Chan School of Public Health, and those of participating registries as required. The patients/participants provided informed consent to participate in the study.

## Author contributions

FA-A: Conceptualization, Data curation, Formal analysis, Writing-Original draft, Writing – review & editing. NS: Conceptualization, Data curation, Writing-Original draft, Writing – review & editing. MT: Data curation, Writing – review & editing. TH: Data curation, Writing – review & editing. KT: Data curation, Writing – review & editing. AV: Data curation, Writing – review & editing. KE: Writing – review & editing. EP: Funding acquisition, Data curation, Writing – review & editing. JH: Data curation, Writing – review & editing. ST: Conceptualization, Funding acquisition, Supervision, Writing – review & editing. BF: Conceptualization, Methodology, Supervision, Writing – review & editing. All authors contributed to the article and approved the submitted version.

## Supplementary material

Supplementary Figure 1 Aggregation plots show all combinations of missing (yellow) and non-missing (blue) values across biomarkers, from the highest to lowest frequency among Invasive epithelial ovarian cancer cases in **(A)** NECC and **(B)** NHS/NHSII. Imputation was conducted using *k*-Nearest Neighbors (*k*NN; k=5) with the *VIM* R package. Data were assumed to be missing at random or completely at random. To avoid scaling issues and ensure comparability across markers and studies, we applied *z*-score transformation. The horizontal bars to the right of the grid show the frequencies of the corresponding combinations, while the vertical bars on top of it present the proportions of missing values in each variable.

Supplementary Figure 2 Box plots represent the distribution of average immunohistochemistry (IHC) scores before (top row) and after (bottom row) applying imputation method among the invasive epithelial ovarian cancer cases in **(A)** NECC and **(B)** NHS/NHSII.

Supplementary Figure 3 Tissue marker associations (odds ratio [OR] and 95% confidence intervals [CI]) with optimal debulking status among invasive epithelial ovarian cancer cases in NHS/NHSII/NECC (n=204) in Type 2 tumors. All models were adjusted for study (NHS/NHSII and NEC). In the logistic regression models 1 = optimally debulked and 0 = sub-optimally debulked. Type 2 tumors include high-grade serous or poorly differentiated, Transitional/Brenner, Carcinosarcoma, high grade mixed histology.

Supplementary Figure 4 Prediction modeling of optimal debulking status using epidemiologic and clinical predictors in Type 2 tumors. Average posterior means and associated average posterior probabilities of epidemiologic and clinical predictors being selected in the final prediction model of optimal debulking status and area under the curve (AUC) of the prediction models using Bayesian model averaging among invasive epithelial ovarian cancer cases (n=537) in NHS/NHSII/NEC. We assessed the posterior probability of 11 predictors for possible selection in the final model and conducted five 5-fold cross-validations. The bar chart **(A)** presents the average posterior mean across the 25 models that were run in total; grey bars denote predictors with average posterior probabilities <20% and yellow bars denote predictors with average posterior probability ≥ 20%. **(B)** presents the five average AUCs from the five 5-fold cross-validations and in the legend overall AUC, which is the average of the average AUCs from the 5-fold CV, is presented. Type 2 tumors include high-grade serous or poorly differentiated, Transitional/Brenner, Carcinosarcoma, high grade mixed histology.

Supplementary Figure 5 Prediction modeling of optimal debulking status using tissue markers in Type 2 tumors. Average posterior means and associated average posterior probabilities of tissue markers being selected in the final prediction model of optimal debulking status and area under the curve (AUC) of the prediction models using Bayesian model averaging among invasive epithelial ovarian cancer cases (n=150) in NHS/NHSII/NEC. We assessed the posterior probability of 24 tissue marker predictors for possible selection in the final model and conducted five 5-fold cross-validations. The bar chart **(A)** presents the average posterior mean across the 25 models that were run in total; grey bars denote predictors with average posterior probabilities <20% and yellow bars denote predictors with average posterior probability ≥ 20%. **(B)** presents the five average AUCs from the five 5-fold cross-validations and in the legend overall AUC, which is the average of the average AUCs from the 5-fold CV, is presented. Type 2 tumors include high-grade serous or poorly differentiated, Transitional/Brenner, Carcinosarcoma, high grade mixed histology.

Supplementary Figure 6 Prediction modeling of optimal debulking status using epidemiologic/clinical predictors and tissue markers in Type 2 tumors. Average posterior means and associated average posterior probabilities of epidemiologic/clinical predictors and tissue markers being selected in the final prediction model of optimal debulking status and area under the curve (AUC) of the prediction models using Bayesian model averaging among invasive epithelial ovarian cancer cases (n=150) in NHS/NHSII/NEC. We assessed the posterior probability of 24 tissue markers in addition to our base model of clinical predict score, which included epidemiologic/clinical variables that had ≥ 20% posterior probability of being selected in the final prediction model of optimal debulking status (i.e. aspirin, pack-years of smoking, and menopausal status), for possible selection in the final model and conducted five 5-fold cross-validations. The bar chart **(A)** presents the average posterior mean across the 25 models that were run in total; grey bars denote predictors with average posterior probabilities <20% and yellow bars denote predictors with average posterior probability ≥ 20%. **(B)** presents the five average AUCs from the five 5-fold cross-validations and in the legend overall AUC, which is the average of the average AUCs from the 5-fold CV, is presented. Type 2 tumors include high-grade serous or poorly differentiated, Transitional/Brenner, Carcinosarcoma, high grade mixed histology.
