# Supplementary figures and images for: Predictors of residual disease after debulking surgery in advanced stage ovarian cancer
Source: Front Oncol. 2023 Jan 24;13:1090092. doi: 10.3389/fonc.2023.1090092 (PMC9902593; doi:10.3389/fonc.2023.1090092)

Supplementary Figure S1

A.

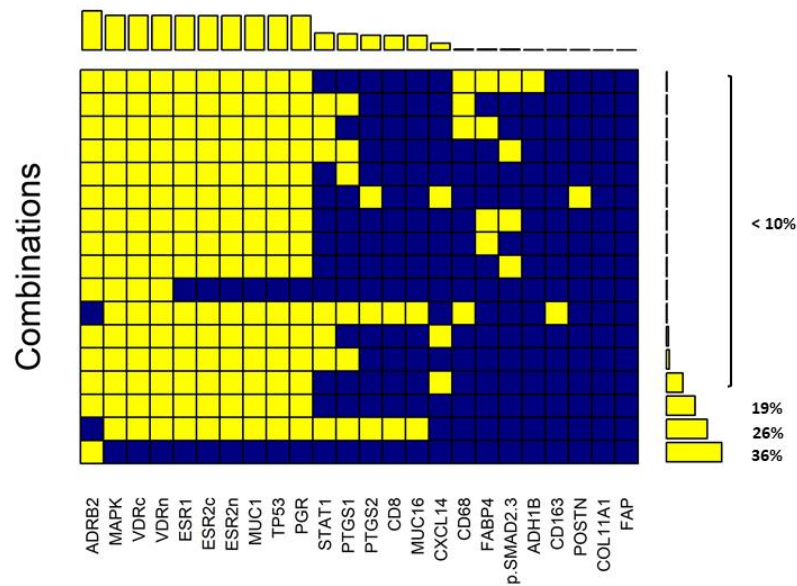

B.

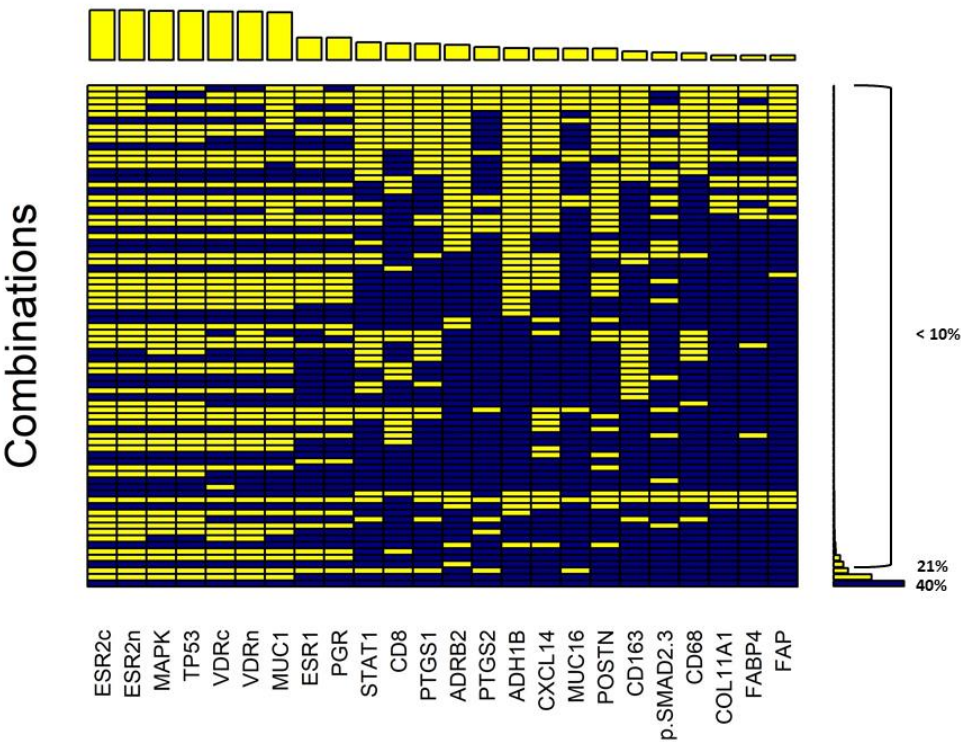

Supplement: Supplementary Figure 1 — Aggregation plots show all combinations of missing (yellow) and non-missing (blue) values across biomarkers, from the highest to lowest frequency among Invasive epithelial ovarian cancer cases in (A) NECC and (B) NHS/NHSII. Imputation was conducted using k-Nearest Neighbors (kNN; k=5) with the VIM R package. Data were assumed to be missing at random or completely at random. To avoid scaling issues and ensure comparability across markers and studies, we applied z-score transformation. The horizontal bars to the right of the grid show the frequencies of the corresponding combinations, while the vertical bars on top of it present the proportions of missing values in each variable. [file DataSheet_2.pdf]

Supplementary Figure S2

A.

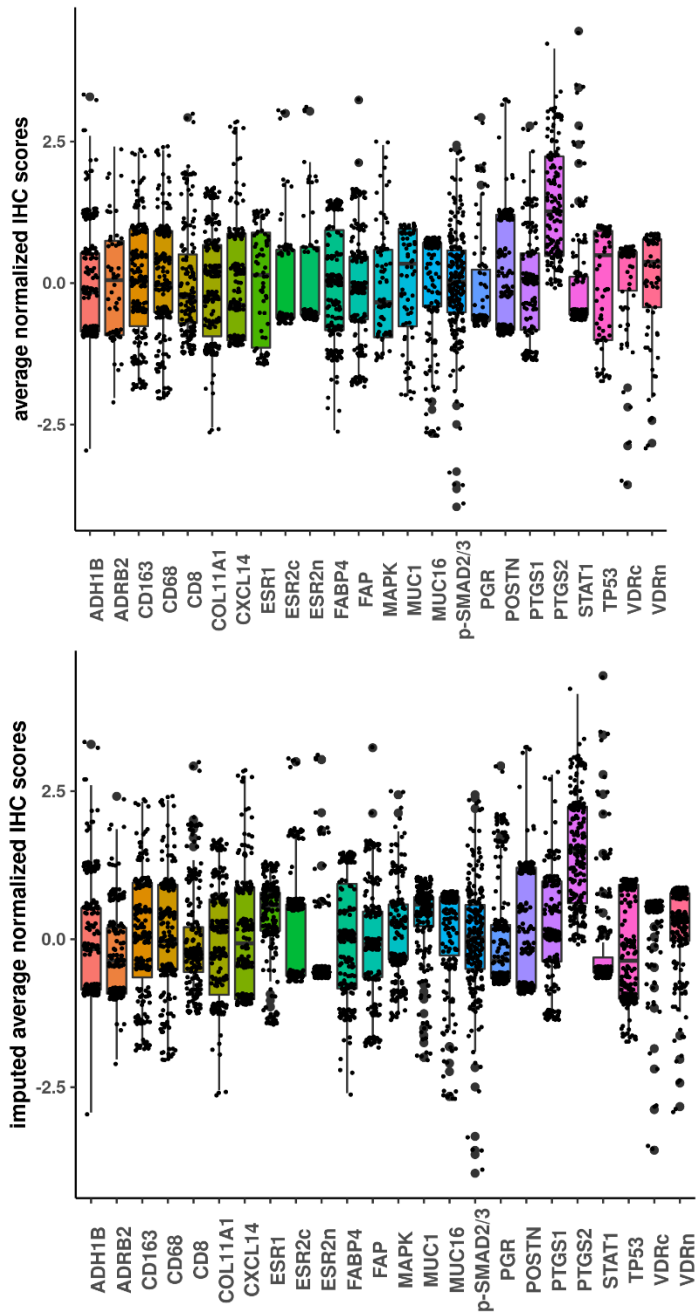

B.

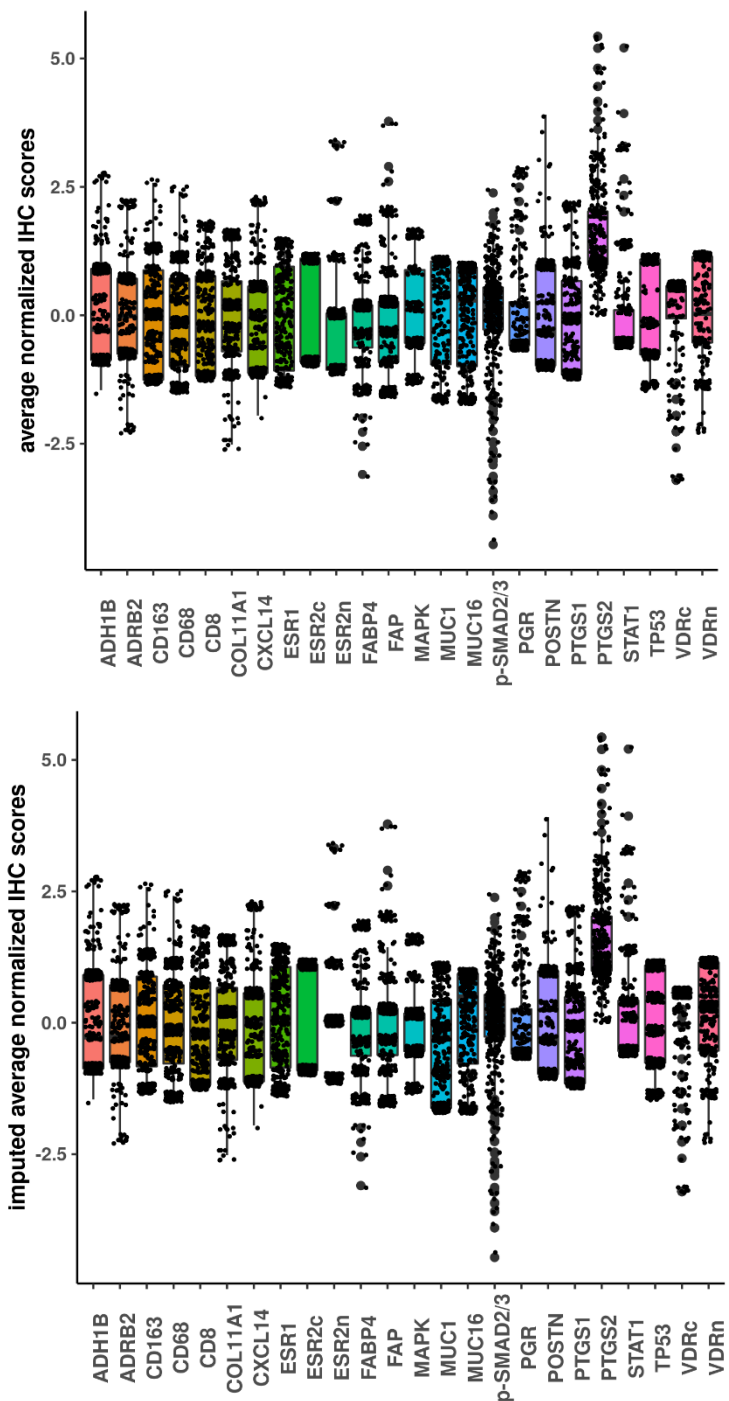

Supplement: Supplementary Figure 2 — Box plots represent the distribution of average immunohistochemistry (IHC) scores before (top row) and after (bottom row) applying imputation method among the invasive epithelial ovarian cancer cases in (A) NECC and (B) NHS/NHSII. [file DataSheet_3.pdf]

Supplementary Figure S3

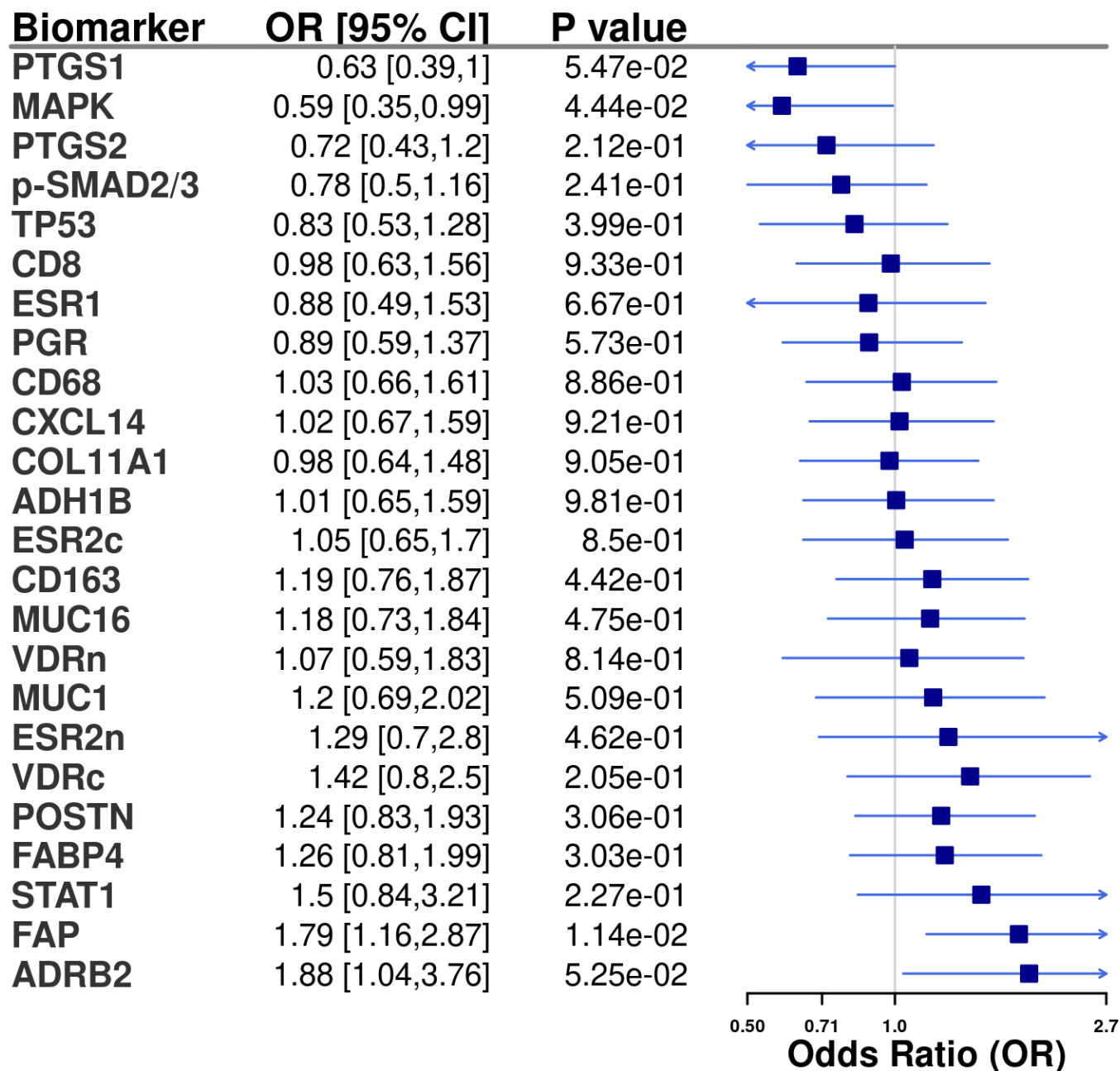

Supplement: Supplementary Figure 3 — Tissue marker associations (odds ratio [OR] and 95% confidence intervals [CI]) with optimal debulking status among invasive epithelial ovarian cancer cases in NHS/NHSII/NECC (n=204) in Type 2 tumors. All models were adjusted for study (NHS/NHSII and NEC). In the logistic regression models 1 = optimally debulked and 0 = sub-optimally debulked. Type 2 tumors include high-grade serous or poorly differentiated, Transitional/Brenner, Carcinosarcoma, high grade mixed histology. [file DataSheet_4.pdf]

Supplementary Figure S4

A.

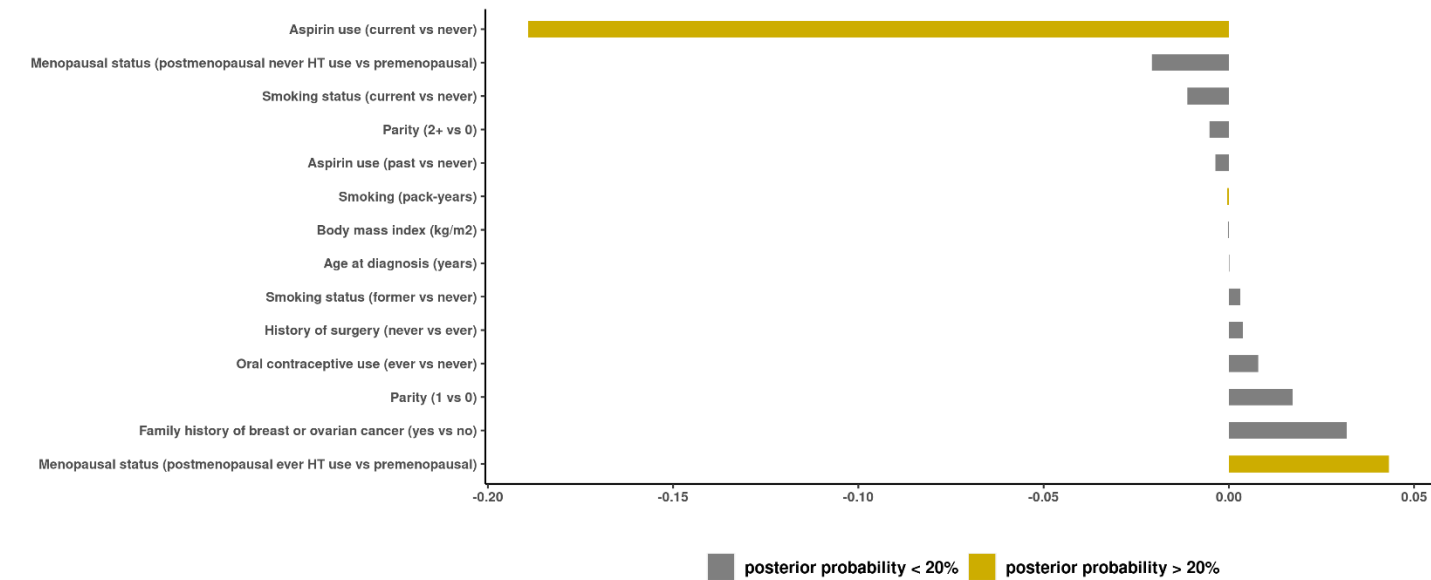

B.

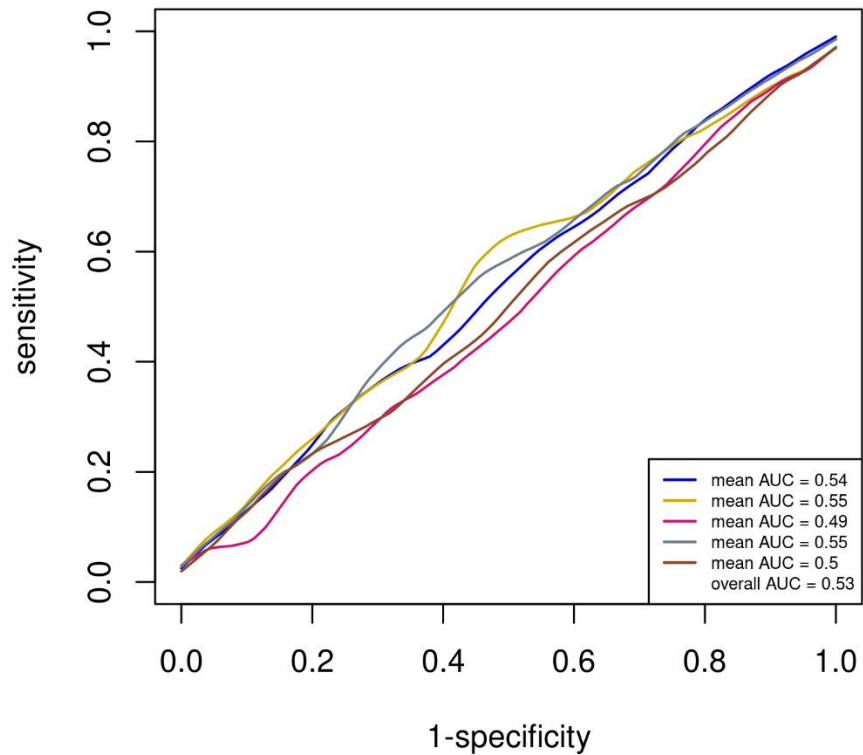

Supplement: Supplementary Figure 4 — Prediction modeling of optimal debulking status using epidemiologic and clinical predictors in Type 2 tumors. Average posterior means and associated average posterior probabilities of epidemiologic and clinical predictors being selected in the final prediction model of optimal debulking status and area under the curve (AUC) of the prediction models using Bayesian model averaging among invasive epithelial ovarian cancer cases (n=537) in NHS/NHSII/NEC. We assessed the posterior probability of 11 predictors for possible selection in the final model and conducted five 5-fold cross-validations. The bar chart (A) presents the average posterior mean across the 25 models that were run in total; grey bars denote predictors with average posterior probabilities <20% and yellow bars denote predictors with average posterior probability ≥ 20%. (B) presents the five average AUCs from the five 5-fold cross-validations and in the legend overall AUC, which is the average of the average AUCs from the 5-fold CV, is presented. Type 2 tumors include high-grade serous or poorly differentiated, Transitional/Brenner, Carcinosarcoma, high grade mixed histology. [file DataSheet_5.pdf]

Supplementary Figure S5

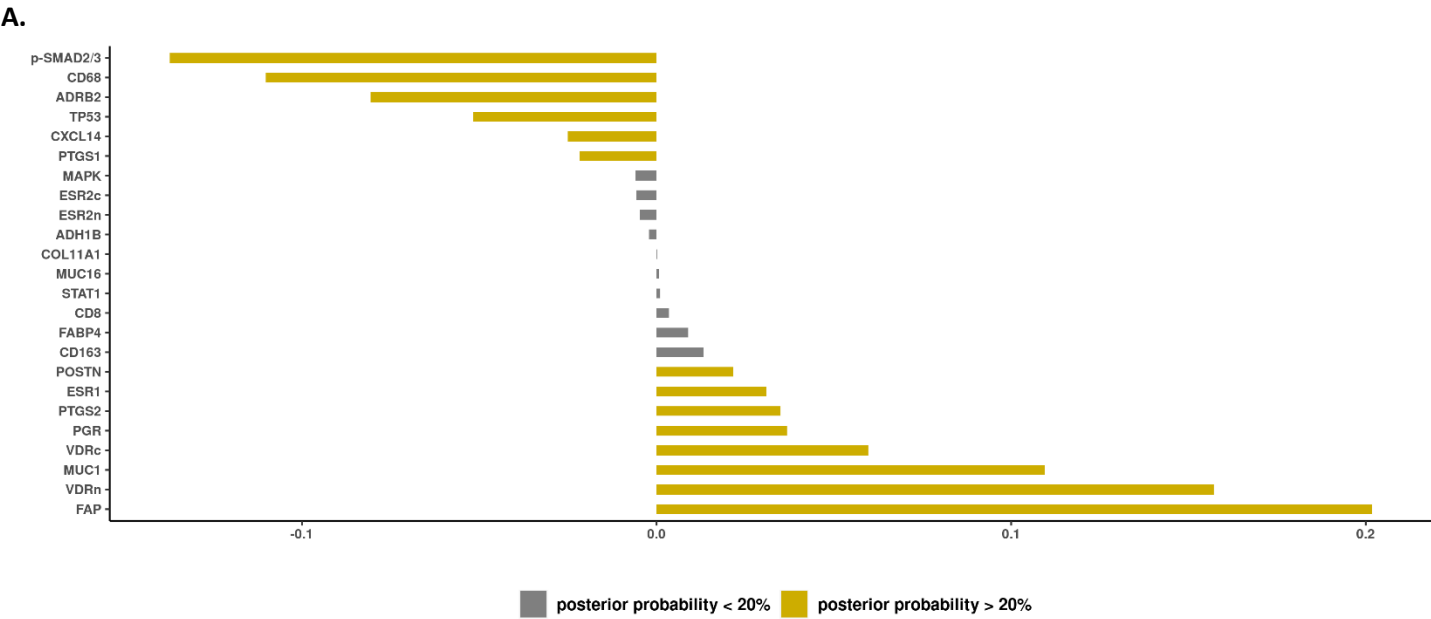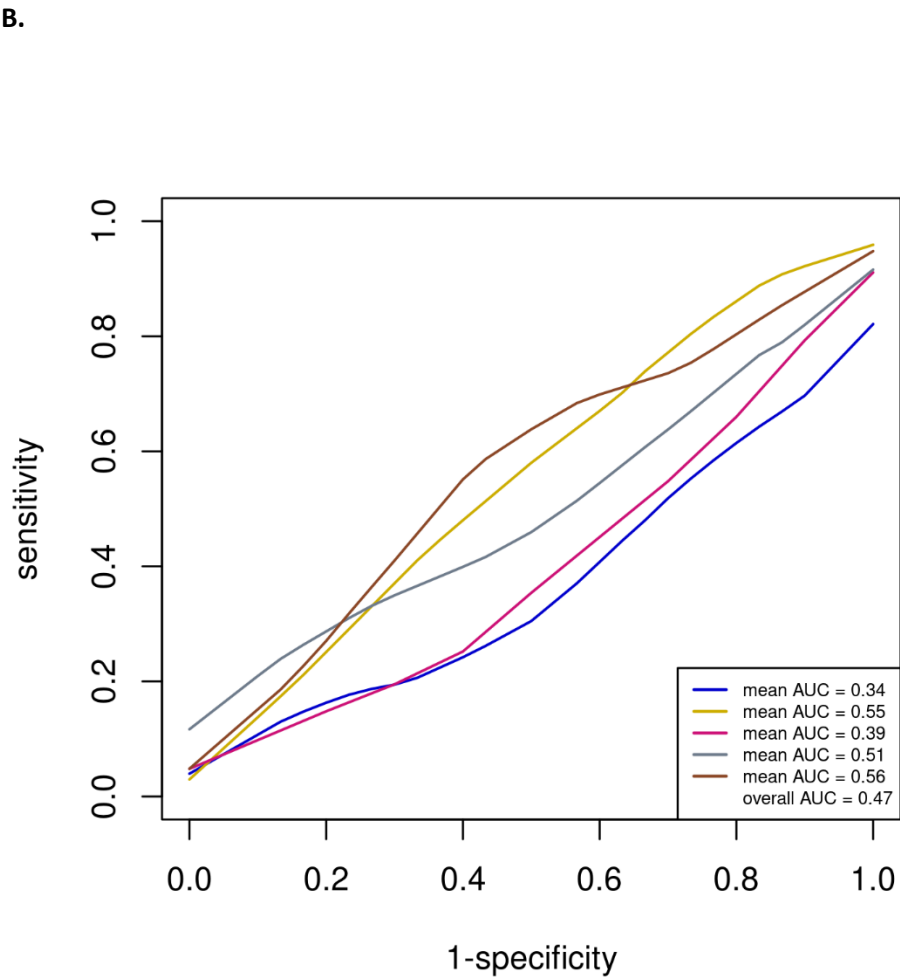

Supplement: Supplementary Figure 5 — Prediction modeling of optimal debulking status using tissue markers in Type 2 tumors. Average posterior means and associated average posterior probabilities of tissue markers being selected in the final prediction model of optimal debulking status and area under the curve (AUC) of the prediction models using Bayesian model averaging among invasive epithelial ovarian cancer cases (n=150) in NHS/NHSII/NEC. We assessed the posterior probability of 24 tissue marker predictors for possible selection in the final model and conducted five 5-fold cross-validations. The bar chart (A) presents the average posterior mean across the 25 models that were run in total; grey bars denote predictors with average posterior probabilities <20% and yellow bars denote predictors with average posterior probability ≥ 20%. (B) presents the five average AUCs from the five 5-fold cross-validations and in the legend overall AUC, which is the average of the average AUCs from the 5-fold CV, is presented. Type 2 tumors include high-grade serous or poorly differentiated, Transitional/Brenner, Carcinosarcoma, high grade mixed histology. [file DataSheet_6.pdf]

Supplementary Figure S6

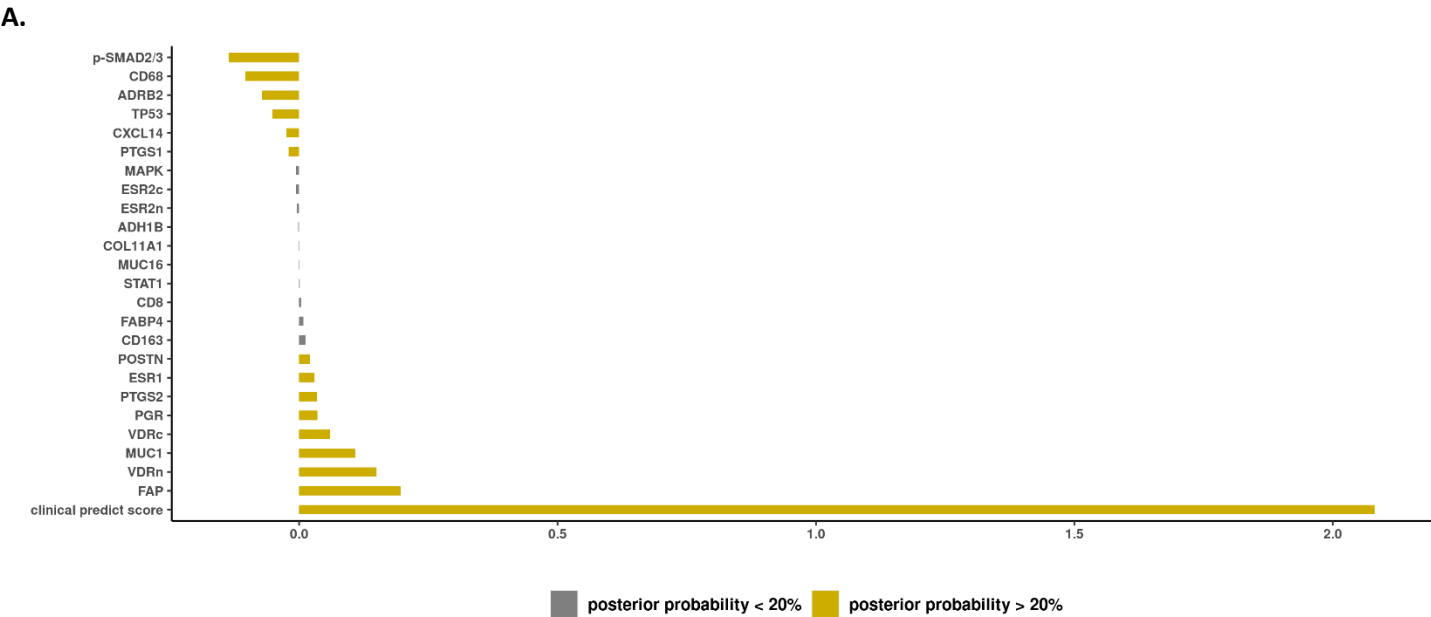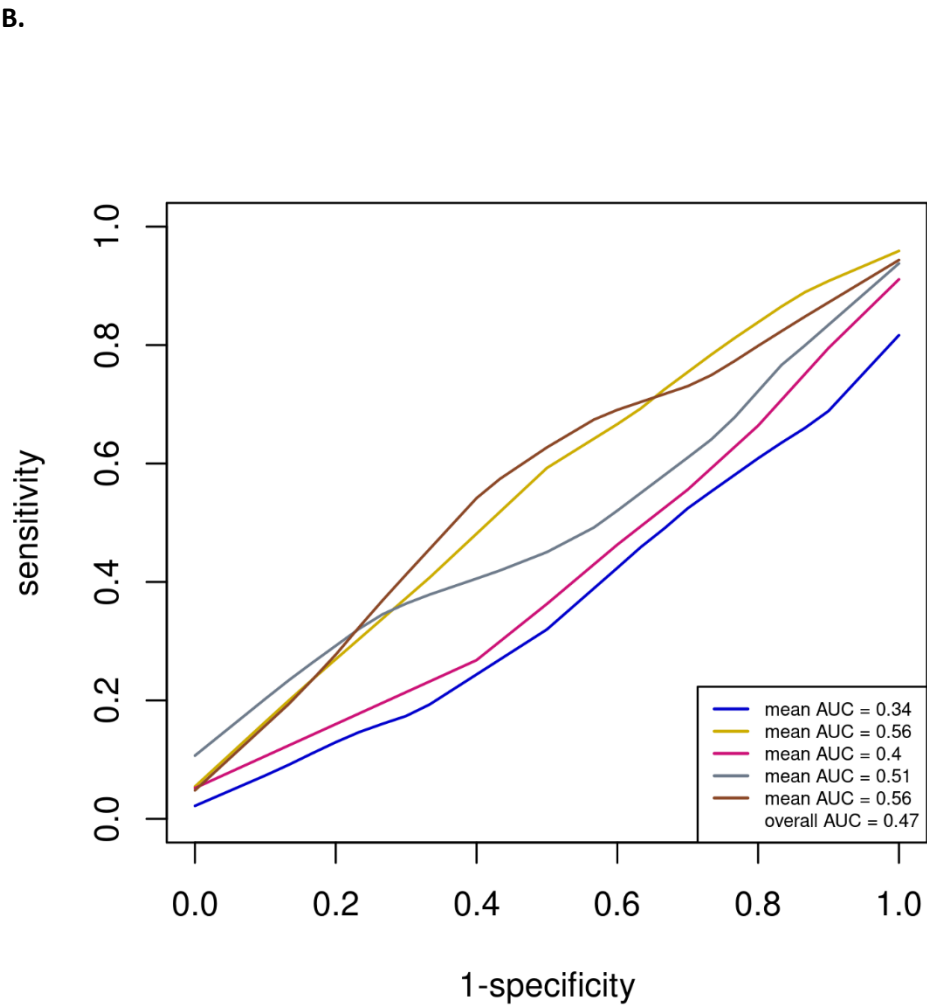

Supplement: Supplementary Figure 6 — Prediction modeling of optimal debulking status using epidemiologic/clinical predictors and tissue markers in Type 2 tumors. Average posterior means and associated average posterior probabilities of epidemiologic/clinical predictors and tissue markers being selected in the final prediction model of optimal debulking status and area under the curve (AUC) of the prediction models using Bayesian model averaging among invasive epithelial ovarian cancer cases (n=150) in NHS/NHSII/NEC. We assessed the posterior probability of 24 tissue markers in addition to our base model of clinical predict score, which included epidemiologic/clinical variables that had ≥ 20% posterior probability of being selected in the final prediction model of optimal debulking status (i.e. aspirin, pack-years of smoking, and menopausal status), for possible selection in the final model and conducted five 5-fold cross-validations. The bar chart (A) presents the average posterior mean across the 25 models that were run in total; grey bars denote predictors with average posterior probabilities <20% and yellow bars denote predictors with average posterior probability ≥ 20%. (B) presents the five average AUCs from the five 5-fold cross-validations and in the legend overall AUC, which is the average of the average AUCs from the 5-fold CV, is presented. Type 2 tumors include high-grade serous or poorly differentiated, Transitional/Brenner, Carcinosarcoma, high grade mixed histology. [file DataSheet_7.pdf]
