# Supplementary material for: Predictors of residual disease after debulking surgery in advanced stage ovarian cancer
Source: Front Oncol. 2023 Jan 24;13:1090092. doi: 10.3389/fonc.2023.1090092 (PMC9902593; doi:10.3389/fonc.2023.1090092)
Supplement: Supplementary file 8 [file Table_1.docx]

**Supplementary Tables**

| **Supplementary Table S1. Details on primary antibodies, dilutions, and retrieval methods** | | | | |
| --- | --- | --- | --- | --- |
| **Antibody** | **Protein name** | **Catalog # (Company)** | **Dilution** | **Retrieval** |
| ADH1B | Alcohol Dehydrogenase 1B | LS-C156085 (Lifespan Bio) | 50 | H2(20min) |
| ADRB2 | Adrenoceptor Beta 2 | HPA003431 (Sigma) | 100 | H1(30min) |
| CD163 | CD163 antigen | VP-C374 (Vector) | 250 | H2 (30 min) |
| CD68 | CD68 antigen | M0876 (Dako) | 200 | H1 (30 min) |
| CD8 | CD8 Antigen | Clone 144B (Dako) | 200 | H2 (15 min) |
| COL11A1 | Collagen Type XI Alpha 1 Chain | HPA008405 (Sigma) | 200 | H1(30min) |
| PTGS1 | Cyclooxygenase-1 | HPA002834 (Sigma Aldrich) | 1:50 | H2 (20 min) |
| PTGS2 | Cyclooxygenase-2 | RM-9121-R7 (Neomarkers) | Ready To Use | H1 (40 min) |
| CXCL14 | C-X-C Motif Chemokine Ligand 14 | ab46010 (abcam) | 1000 | H1(30min) |
| ESR1 | Estrogen receptor alpha | primary antibody: rabbit monoclonal; clone SP1 (Neomarkers) | 40 |  |
| ESR2c | Estrogen receptor beta cytoplasmic stain | anti-estrogen receptor beta 1, clone: PPG5/10 (Thermo Scientific Pierce) |  |  |
| ESR2n | Estrogen receptor beta nuclear stain | anti-estrogen receptor beta 1, clone: PPG5/10 (Thermo Scientific Pierce) |  |  |
| FABP4 | Fatty Acid Binding Protein 4 | ab13979 (abcam) | 200 | H1(30min) |
| FAP | Fibroblast Activation Protein Alpha | ab53066 (abcam) | 100 | H2(20min) |
| MAPK | Mitogen-Activated Protein Kinase | rabbit monocloncal; p-ERK (Cell Signaling Technologies) | 150 |  |
| MUC1 | Mucin 1 | VP-M654, clone MA552 (Vector) | 600 |  |
| MUC16 | Mucin 16 | clone M11 (DAKO) | 100 |  |
| TP53 | Tumor Protein P53 | mouse monoclonal; clone DO-1 (Beckman Coulter) | 500 |  |
| POSTN | Periostin | RD181045050 (BioVendor) | 500 | H1(30min) |
| PGR | Progesterone receotor | mouse monoclonal; clone PgR 636 (Dako) | 150 |  |
| pSmad2/3 | Phospho-Smad2/3 | 3101 (CST) | 1000 | H1(30min) |
| STAT1 | Signal Transducer And Activator Of Transcription 1 | 31369 (abcam) | 100 | H2(20min) |
| VDRc | Vitamin D receptor cytoplasmic stain | sc-13133 Clone D-6 (Santa Cruz) | 400 | H1(30min) |
| VDRn | Vitamin D receptor nuclear stain | NBP1-19478 (Novus) | 500 | H2(20min) |
| *Antigen retrieval was performed online using Bond Epitope Retrieval 1 ("H1") pH 6.0, or Bond Epitope Retrieval 2 ("H2") pH 8.0 at 100°C for the time indicated. | | | | |

**Supplementary Table S2**: Distribution of demographic/clinical characteristics of advanced stage invasive ovarian cancer cases with and without debulking status

|  | **Total**  **(n=2045)** | **With data on debulking**  **(n=593)** | **Missing data on debulking**  **(n=1452)** |
| --- | --- | --- | --- |
| **Age,** Mean (SD) | 58.3 (13.8) | 56.9 (12.6) | 58.9 (14.2) |
| **BMI,** Mean (SD) | 26.4 (5.8) | 26.2 (5.7) | 26.5 (5.9) |
| **Smoking status**, n (%) |  |  |  |
| never | 944 (46.2%) | 270 (45.5%) | 674 (46.4%) |
| current | 296 (14.5%) | 87 (14.7%) | 209 (14.4%) |
| former | 805 (39.4%) | 236 (39.8%) | 569 (39.2%) |
| **Smoking (pack-years),** |  |  |  |
| Mean (SD) | 12.4 (19.6) | 11.7 (19.6) | 12.7 (19.5) |
| **Aspirin,** n (%) |  |  |  |
| never | 990 (48.4%) | 391 (65.9%) | 599 (41.3%) |
| current | 602 (29.4%) | 93 (15.7%) | 509 (35.1%) |
| past | 453 (22.2%) | 109 (18.4%) | 344 (23.7%) |
| **Oral Contraceptive use**, n (%) |  |  |  |
| never | 1049 (51.3%) | 294 (49.6%) | 755 (52.0%) |
| ever | 995 (48.7%) | 299 (50.4%) | 696 (47.9%) |
| missing | 1 (<0.1%) | 0 (0%) | 1 (0.1%) |
| **Parity,** n (%) |  |  |  |
| 0 | 319 (15.6%) | 122 (20.6%) | 197 (13.6%) |
| 1 | 200 (9.8%) | 60 (10.1%) | 140 (9.6%) |
| 2 + | 1526 (74.6%) | 411 (69.3%) | 1115 (76.8%) |
| **Menopausal status,** n (%) |  |  |  |
| premenopausal | 468 (22.9%) | 155 (26.1%) | 313 (21.6%) |
| postmenopausal  never used PMH | 691 (33.8%) | 242 (40.8%) | 449 (30.9%) |
| postmenopausal  ever used PMH | 886 (43.3%) | 196 (33.1%) | 690 (47.5%) |
| **Family history of breast or ovarian cancer**, n (%) |  |  |  |
| no | 1739 (85.0%) | 512 (86.3%) | 1227 (84.5%) |
| yes | 306 (15.0%) | 81 (13.7%) | 225 (15.5%) |
| **History of surgery ^a^**, n (%) |  |  |  |
| ever | 753 (36.8%) | 206 (34.7%) | 547 (37.7%) |
| never | 1292 (63.2%) | 387 (65.3%) | 905 (62.3%) |

**Abbreviations**: BMI: Body mass index; CI: Confidence interval; NHS: Nurses’ Health Study; NHSII: Nurses’ Health Study II; NEC: New England Case-Control Study; OR: Odds ratio; PMH: postmenopausal hormone use.

All models were adjusted for study sites (NHS/NHSII and NEC). Odds ratios represent the odds of optimally debulked surgery.

(a) Cases with history of hysterectomy or tubal ligation or Cesarean section.

**Supplementary Table S3:** Association between demographic/clinical characteristics and optimal debulking status among advanced stage invasive epithelial ovarian cancer cases in NHS/NHSII/NEC restricted to Type 2 tumors. In the logistic regression models 1 = optimally debulked and 0 = sub-optimally debulked.

|  | **Total**  **(n=537)** | **Optimally debulked**  **(n=419)** | **Sub-optimally**  **debulked**  **(n=118)** | **OR (95% CI)** |
| --- | --- | --- | --- | --- |
| **Age at diagnosis (years),** Mean (SD) | 57.2 (12.4) | 57.3 (12.3) | 57.0 (12.7) | 1.00 (0.99 to 1.02) |
| **BMI (kg/m^2^),** Mean (SD) | 26.1 (5.6) | 26.0 (5.6) | 26.3 (5.7) | 0.99 (0.96 to 1.03) |
| **Smoking status**, n (%) |  |  |  |  |
| never | 236 (43.9%) | 185 (44.2%) | 51 (43.2%) | 1 (ref.) |
| current | 81 (15.1%) | 60 (14.3%) | 21 (17.8%) | 0.79 (0.44 to 1.44) |
| former | 220 (41.0%) | 174 (41.5%) | 46 (39.0%) | 1.04 (0.67 to 1.64) |
| **Smoking (pack-years),**  Mean (SD) | 12.0 (19.9) | 11.4 (19.4) | 14.2 (21.6) | 0.99 (0.98 to 1.00) |
| **Aspirin,** n (%) |  |  |  |  |
| never | 356 (66.3%) | 288 (68.7%) | 68 (57.6%) | 1 (ref.) |
| current | 81 (15.1%) | 54 (12.9%) | 27 (22.9%) | 0.47 (0.28 to 0.81) |
| past | 100 (18.6%) | 77 (18.4%) | 23 (19.5%) | 0.79 (0.47 to 1.37) |
| **Oral Contraceptive use**, n (%) |  |  |  |  |
| never | 265 (49.3%) | 202 (48.2%) | 63 (53.4%) | 1 (ref.) |
| ever | 272 (50.7%) | 217 (51.8%) | 55 (46.6%) | 1.23 (0.82 to 1.86) |
| **Parity,** n (%) |  |  |  |  |
| 0 | 105 (19.6%) | 82 (19.6%) | 23 (19.5%) | 1 (ref.) |
| 1 | 55 (10.2%) | 46 (11.0%) | 9 (7.6%) | 1.43 (0.63 to 3.51) |
| 2 + | 377 (70.2%) | 291 (69.5%) | 86 (72.9%) | 0.95 (0.55 to 1.58) |
| **Menopausal status,** n (%) |  |  |  |  |
| premenopausal | 130 (49.3%) | 101 (24.1%) | 29 (24.6%) | 1 (ref.) |
| postmenopausal  never used PMH | 223 (41.5%) | 173 (41.3%) | 50 (42.4%) | 0.99 (0.59 to 1.66) |
| postmenopausal  ever used PMH | 184 (34.3%) | 145 (34.6%) | 39 (33.1%) | 1.07 (0.62 to 1.83) |
| **Family history of breast or ovarian cancer**, n (%) |  |  |  |  |
| no | 463 (86.2%) | 357 (85.2%) | 106 (89.8%) | 1 (ref.) |
| yes | 74 (13.8%) | 62 (14.8%) | 12 (10.2%) | 1.53 (0.82 to 3.09) |
| **History of surgery ^a^**, n (%) |  |  |  |  |
| ever | 195 (36.3%) | 150 (35.8%) | 45 (38.1%) | 1 (ref.) |
| never | 342 (63.7%) | 269 (64.2%) | 73 (61.9%) | 1.11 (0.72 to 1.68) |

**Abbreviations**: BMI: Body mass index; CI: Confidence interval; NHS: Nurses’ Health Study; NHSII: Nurses’ Health Study II; NEC: New England Case-Control Study; OR: Odds ratio; PMH: postmenopausal hormone use.

All models were adjusted for study sites (NHS/NHSII and NEC). Odds ratios represent the odds of optimally debulked surgery.

(a) Cases with history of hysterectomy or tubal ligation or Cesarean section.

**Supplementary Table S4**: Distribution of demographic/clinical characteristics and optimal debulking status among advanced stage invasive epithelial ovarian cancer cases on ovarian TMAs in NHS/NHSII/NEC.

|  | **Total**  **(n=166)** | **Optimally debulked**  **(n=135)** | **Sub-optimally**  **debulked**  **(n=31)** |
| --- | --- | --- | --- |
| **Age at diagnosis,** Mean (SD) | 58.4 (11.8) | 58.6 (11.3) | 57.3 (13.8) |
| **BMI,** Mean (SD) | 25.7 (5.0) | 25.9 (5.0) | 24.7 (5.1) |
| **Smoking status**, n (%) |  |  |  |
| never | 84 (50.6%) | 69 (51.1%) | 15 (48.4%) |
| current | 26 (15.7%) | 22 (16.3%) | 4 (12.9%) |
| former | 56 (33.7%) | 44 (32.6%) | 12 (38.7%) |
| **Smoking (pack-years),**  Mean (SD) | 11.7 (20.9) | 10.1 (18.1) | 18.4 (29.7) |
| **Aspirin,** n (%) |  |  |  |
| never | 110 (66.3%) | 91 (67.4%) | 19 (61.3%) |
| current | 22 (13.3%) | 16 (11.9%) | 6 (19.4%) |
| former | 34 (20.5%) | 28 (20.7%) | 6 (19.4%) |
| **Oral Contraceptive use**, n (%) |  |  |  |
| never | 83 (50%) | 69 (51.1%) | 14 (45.2%) |
| ever | 83 (50%) | 66 (48.9%) | 17 (54.8%) |
| **Parity,** n (%) |  |  |  |
| 0 | 34 (20.5%) | 26 (19.3%) | 8 (25.8%) |
| 1 | 16 (9.6%) | 11 (8.1%) | 5 (16.1%) |
| 2 + | 116 (69.9%) | 98 (72.6%) | 18 (58.1%) |
| **Menopausal status,** n (%) |  |  |  |
| premenopausal | 35 (21.1%) | 25 (18.5%) | 10 (32.3%) |
| postmenopausal  never used PMH | 77 (46.4%) | 68 (50.4%) | 9 (29.0%) |
| postmenopausal  ever used PMH | 54 (32.5%) | 42 (31.1%) | 12 (38.7%) |
| **Family history of breast or ovarian cancer** , n (%) |  |  |  |
| no | 143 (86.1%) | 115 (85.2%) | 28 (90.3%) |
| yes | 23 (13.9%) | 20 (14.8%) | 3 (9.7%) |
| **History of surgery ^a^**, n (%) |  |  |  |
| ever | 67 (40.4%) | 54 (40.0%) | 13 (41.9%) |
| never | 99 (59.6%) | 81 (60.0%) | 18 (58.1%) |
| **Tumor Type ^b^**, n (%) |  |  |  |
| type 1 | 16 (9.6%) | 15 (11.1%) | 1 (3.2%) |
| type 2 | 150 (90.4%) | 120 (88.9%) | 30 (96.8%) |

**Abbreviations**: BMI: Body mass index; CI: Confidence interval; NHS: Nurses’ Health Study; NHSII: Nurses’ Health Study II; NEC: New England Case-Control Study; OR: Odds ratio; PMH: postmenopausal hormone use.

All models were adjusted for study sites (NHS/NHSII and NEC). Odds ratios represent the odds of optimally debulked surgery.

(a) Cases with history of hysterectomy or tubal ligation or Cesarean section.

(b) Type 1 tumors: low-grade serous, mucinous, endometrioid, clear cell, low grade mixed; type 2 tumors: high-grade serous or poorly differentiated, Transitional/Brenner, Carcinosarcoma, high grade mixed.

**Supplementary Table S5**: Distribution of demographic/clinical characteristics and optimal debulking status among advanced stage invasive epithelial ovarian cancer cases on ovarian TMAs in NHS/NHSII/NEC restricted to Type 2 tumors.

|  | **Total**  **(n=150)** | **Optimally debulked**  **(n=120)** | **Sub-optimally**  **debulked**  **(n=30)** |
| --- | --- | --- | --- |
| **Age,** Mean (SD) | 58.0 (11.8) | 58.0 (11.4) | 57.9 (13.7) |
| **BMI,** Mean (SD) | 25.5 (4.9) | 25.9 (4.9) | 24.9 (5.0) |
| **Smoking status**, n (%) |  |  |  |
| never | 75 (50.0%) | 61 (50.8%) | 14 (46.7%) |
| current | 24 (16.0%) | 20 (16.7%) | 4 (13.3%) |
| former | 51 (34.0%) | 39 (32.5%) | 12 (40.0%) |
| **Smoking (pack-years),**  Mean (SD) | 11.7 (21.4) | 9.9 (18.3) | 19.0 (30.0) |
| **Aspirin,** n (%) |  |  |  |
| never | 101 (67.3%) | 83 (69.2%) | 18 (60.0%) |
| current | 17 (11.3%) | 11 (9.2%) | 6 (20.0%) |
| past | 32 (21.3%) | 26 (21.7%) | 6 (20.0%) |
| **Oral Contraceptive use**, n (%) |  |  |  |
| never | 74 (49.3%) | 60 (50.0%) | 14 (46.7%) |
| ever | 76 (50.7%) | 60 (50.0%) | 16 (53.3%) |
| **Parity,** n (%) |  |  |  |
| 0 | 29 (19.3%) | 22 (18.3%) | 7 (23.3%) |
| 1 | 14 (9.3%) | 9 (7.5%) | 5 (16.7%) |
| 2 + | 107 (71.3%) | 89 (74.2%) | 18 (60.0%) |
| **Menopausal status,** n (%) |  |  |  |
| premenopausal | 32 (21.3%) | 23 (19.2%) | 9 (30.0%) |
| postmenopausal  never used PMH | 69 (46.0%) | 60 (50.0%) | 9 (30.0%) |
| postmenopausal  ever used PMH | 49 (32.7%) | 37 (30.8%) | 12 (40.0%) |
| **Family history of breast or ovarian cancer** , n (%) |  |  |  |
| no | 130 (86.7%) | 103 (85.8%) | 27 (90.0%) |
| yes | 20 (13.3%) | 17 (14.2%) | 3 (10.0%) |
| **History of surgery ^a^**, n (%) |  |  |  |
| ever | 64 (42.7%) | 51 (42.5%) | 13 (43.3%) |
| never | 86 (57.3%) | 69 (57.5%) | 17 (56.7%) |

**Abbreviations**: BMI: Body mass index; CI: Confidence interval; NHS: Nurses’ Health Study; NHSII: Nurses’ Health Study II; NEC: New England Case-Control Study; OR: Odds ratio; PMH: postmenopausal hormone use.

All models were adjusted for study sites (NHS/NHSII and NEC). Odds ratios represent the odds of optimally debulked surgery.

(a) Cases with history of hysterectomy or tubal ligation or Cesarean section.
